# Deep Learning for Echocardiography: Introduction for Clinicians and Future Vision: State-of-the-Art Review

**DOI:** 10.3390/life13041029

**Published:** 2023-04-17

**Authors:** Chayakrit Krittanawong, Alaa Mabrouk Salem Omar, Sukrit Narula, Partho P. Sengupta, Benjamin S. Glicksberg, Jagat Narula, Edgar Argulian

**Affiliations:** 1 Cardiology Division, NYU Langone Health, NYU School of Medicine, New York, NY 10016, USA; 2 Icahn School of Medicine at Mount Sinai, Mount Sinai Heart, New York, NY 10029, USA; 3 Division of Cardiovascular Medicine, Icahn School of Medicine at Mount Sinai Morningside, Mount Sinai Heart, New York, NY 10029, USA; 4 Department of Medicine, Yale School of Medicine, New Haven, CT 06512, USA; 5 Robert Wood Johnson University Hospital, Rutgers Robert Wood Johnson Medical School, New Brunswick, NJ 08901, USA; 6 Hasso Plattner Institute for Digital Health, Icahn School of Medicine at Mount Sinai, New York, NY 10029, USA

**Keywords:** deep learning, artificial intelligence, echocardiography

## Abstract

Exponential growth in data storage and computational power is rapidly narrowing the gap between translating findings from advanced clinical informatics into cardiovascular clinical practice. Specifically, cardiovascular imaging has the distinct advantage in providing a great quantity of data for potentially rich insights, but nuanced interpretation requires a high-level skillset that few individuals possess. A subset of machine learning, deep learning (DL), is a modality that has shown promise, particularly in the areas of image recognition, computer vision, and video classification. Due to a low signal-to-noise ratio, echocardiographic data tend to be challenging to classify; however, utilization of robust DL architectures may help clinicians and researchers automate conventional human tasks and catalyze the extraction of clinically useful data from the petabytes of collected imaging data. The promise is extending far and beyond towards a contactless echocardiographic exam—a dream that is much needed in this time of uncertainty and social distancing brought on by a stunning pandemic culture. In the current review, we discuss state-of-the-art DL techniques and architectures that can be used for image and video classification, and future directions in echocardiographic research in the current era.

## 1. Introduction

**Figure 1:**
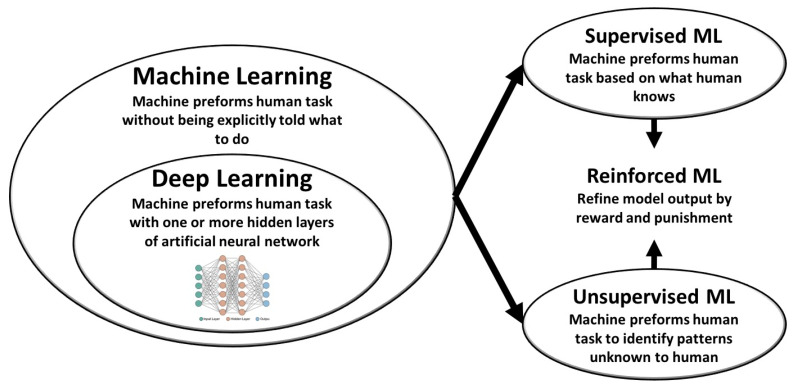
Machine learning (ML) as a part of artificial intelligence applications. Artificial intelligence (AI) is the ability of a computer to perform tasks commonly associated with intelligent beings. ML is a diverse and rich field of science designed to imitate human capabilities. The types of machine learning include ML and DL algorithms that can perform tasks in supervised and unsupervised fashions, and reinforcement learning algorithms can be incorporated to refine model outputs using reward and punishment systems.

Artificial intelligence (AI) has facilitated our capabilities of handling large-scale multi-faceted data. AI is involved in several scientific and non-scientific fields of life for data processing, and it has transformed our lives fundamentally in many fields such as image processing, voice recognition systems, and complex strategy games [1]. In the clinical arena, AI has the potential to outperform conventional analyses with reduction of cost, cognitive errors, and the intra- and inter-observer variability. In medicine, AI can help us in two complementary directions towards a medical and clinical paradigm shift: first, automation of labor-demanding conventional human tasks; and, second, disease phenotyping and big data modelling for better personalized risk stratification and newer classification of disease. Although any medical data are virtually fit for training AI algorithms, and efforts to apply machine learning (ML) to medical imaging in particular, have shown promise in computer-assisted diagnosis. Deep learning (DL) is a subset of ML suitable for large datasets and particularly images by automatically learning and constructing variables and feature representations of a set of data. DL has paved the path for breakthroughs in the way we handle medical imaging data [2,3,4,5,6,7]. DL involves more complex levels of data handling and processing than traditional ML (Figure 1), making it more suitable for image and video analytics (Table 1). In the heart of medical imaging in cardiovascular medicine, echocardiography is a uniquely well-suited approach for the application of DL in cardiology. We have previously shown how data retrieved from echocardiography are well suited for training DL algorithms in a manner that is not any different from any data currently used to train DL algorithms in other fields of computer science [8,9].

DL has attracted attention recently in the field of echocardiography and cardiovascular imaging. The concepts of video classification are well known to researchers in the computer science field but are relatively new in medicine. Recent studies have examined the applications of DL in echocardiography, specifically addressing diagnosis and classification in the assessment of cardiac anatomy [10], diastolic dysfunction [11], left ventricular chamber size, strain and function, wall thickness [12,13,14,15,16], global and regional function [17], mitral regurgitation (MR) severity [18], congenital heart disease detection in the fetus [19], and shunt detection in pediatrics [20], as well as automatic detection of myocardial speckle patterns [21], among others. In addition, studies have examined the applications of DL in echocardiography in the pediatric population [22,23] and congenital heart disease [24]. Most importantly, recent studies have demonstrated that DL could be used in echo-assisted advanced heart failure intervention (e.g., real-time detection of aortic valve opening in LVAD patients, and post-operative right ventricular failure) [25,26]. However, the terms used in the field of computer science, namely ‘pattern recognition’, ‘computer vision’, ‘video classification’, ‘YOLO algorithm’, ‘supervised and unsupervised learning’, ‘artificial neuron’, ‘layer’, ‘pooling’, and ‘convolution’, are still foreign for most clinicians. The aims of the current review are twofold: firstly, to introduce the basic concepts of DL and to explain its relevance to echocardiographic imaging. Secondly, to provide examples of DL applications in the echocardiographic laboratory for clinicians and scientists. In the post-pandemic era and the next emerging pandemic, we explore the promise DL holds for the future of the echocardiographic laboratory where contactless echocardiographic exams are becoming more and more necessary.

## 2. Core Fundamental Concepts of DL

DL, as a subdivision of ML, uses layered structure algorithms inspired from the human brain called neural networks [27]. Briefly, neural networks consists of a series of layers of nodes and edges representing data entering and the complex interactions between them. Layers of nodes within a neural network are not physical structures but successive steps in an analytical algorithm. Neural networks are trained by identifying patterns in the input dataset to produce useful predictions in the output layer. During the training process, certain patterns are attempted to be captured from input data and the other hidden layers before reaching output. The parameters are progressively tuned via a loss function in each hidden layer until the process results in as good predictions as possible. Once a model is trained it then is subsequently used to make predictions on new, unseen data. An artificial neural network (ANN) as a concept has led to tweaks of newer algorithms that have leveraged the field of DL in image processing and video classification (Table 2), and variants and combinations of these algorithms (hybrid models) are occasionally used to establish refined results [28,29]. 

In the process of introducing clinicians to DL to facilitate the usability of these resources in medicine, one should be familiar with terms and frameworks commonly used in computer science. In this section we aim to introduce a simplified framework of the core concepts used in the field of DL and take the reader on a journey from defining the building blocks of an algorithm all the way towards the complete model and the processing codes that are used to handle data. This section is not intended to be an exhaustive explanation of these concepts but rather just an oversimplified introduction intended for clinicians to break the illiteracy. 

### 2.1. What Are the Components of a DL Model?

**Figure 2:**
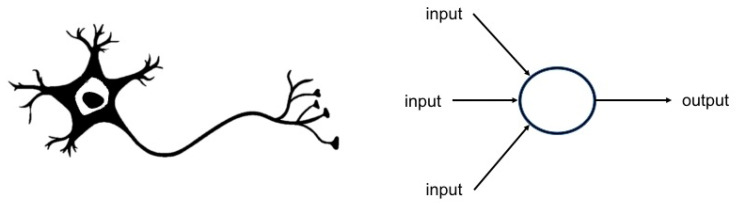
The basic structure of ‘artificial neurons’. The ‘artificial neuron’ (units, right panel) is similar in structure to the neuron in neurobiology in that it can receive input information, process it, and forward it as output information for further processing.

DL models are composed of two main building elements: neurons and layers. The most basic structure in DL (the unit) is the artificial neuron. Artificial neurons usually have several incoming and outgoing connections. The term ‘artificial neuron’ implies an anatomical and functional similarity to neurons in biology in the way they process new information and generate outputs (Figure 2).

**Figure 3:**
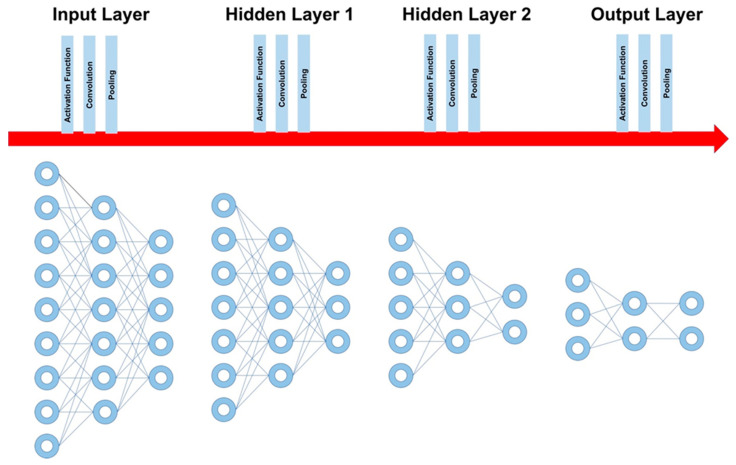
Deep learning layers. A layer is the building block in DL and is composed of neurons that receive weighted input and transform it into an output, which is usually passed to the next layer. In each layer, several processing functions and filters can be applied; however, each layer should be uniform in terms of these functions (e.g., pooling and convolution). The first layer of a model is called the input layer, and the last layer is called the output layer, and all layers in between are called hidden layers (the processing layers).

Neurons, as the most basic building units, are then organized and rearranged in forming layers of neurons. A layer within DL is the unit container that receives weighted input and transforms it into an output, which is usually passed to the next layer. Neurons within each layer are uniformly processed in terms of activation function, pooling, convolution, etc. (Figure 3). The most basic DL algorithm consists of three layers of neurons: one initial layer (input layer) composed of neurons carrying raw data variables, and one middle layer (hidden layer) that is composed of neurons whose function is to process the incoming data and pass them to the final layer (output layer), which contains the final data and reduced variables that represent the final model output. Most importantly, the middle “hidden” part of the algorithm can be composed of one or more layers based on the sophistication of the model and the nature of the data, and, as the name implies, hidden layers are much less interpretable, unlike the input and output layers.

In summary, a layer is the building block in DL and is composed of multiple neurons being processed uniformly in each layer. The basic DL model is composed of three layers (Figure 3): the first layer of a model is called the input layer, the last layer is called the output layer, and all layers in between are called hidden layers (the processing layers) where tasks are performed on the incoming data and passed to the next layer. 

### 2.2. How Are the Data Processed from Layer to Layer?

There are several stages of processing once the data are fed into the algorithm. These stages range from simple mathematical tasks, such as calculating weights and biases, to handling the direction of processing (forward and backward), untwining several aspects in the complicated raw data such as medical images (convolution), allowing for separate processing of each element, and potentially reducing these elements to the most important ones (pooling). Here we provide simple explanations on each of these aspects of the DL algorithm processing journey.

*Weight, bias, and activation functions:* the mathematical journey of information within and between units of neural networks from input to output includes calculation of weight and bias, and application of activation functions. If a unit has more than one input, a “*weight*” that represents the importance of each of these inputs for the neuron is assigned. These weights are updated as the model learns the data until, finally, a higher weight is assigned to inputs that are more important compared with the ones that are considered less important. The result of all weights is then multiplied by the input, and then another linear function called “bias” is added to change the range of weights to produce the final linear outcome. Finally, a non-linear function is added to the final component by applying the “activation function”. Activation functions from a sigmoid function (generates a smooth output between 0 and 1 suitable for binary data) [30] and rectifies linear units (ReLU) [31] to Softmax for output prediction (similar to sigmoid function but it is more suitable for multinomial classification problems) [32]. In general, modern approaches use a main function choice for the entire neural network. Now, there are several purposed robust activation functions, but ReLU appears to be the most commonly used [33,34]. 

*Forward and backward propagation:* as the name implies, in forward propagation (or forward feed) the information travels in a single direction, that is from the input layer through the hidden layers to the output layer, until a final output is generated without any backward movement through the model. In backward propagation, the output of a specific layer can be back fed to the same or previous layers after calculation of error to update the weights of the network and reduce the error. In addition, backward feed can be used to study temporal events, a property that makes it suitable for assessment of echocardiographic videos, and the events and values, which significantly vary with time. 

**Figure 4:**
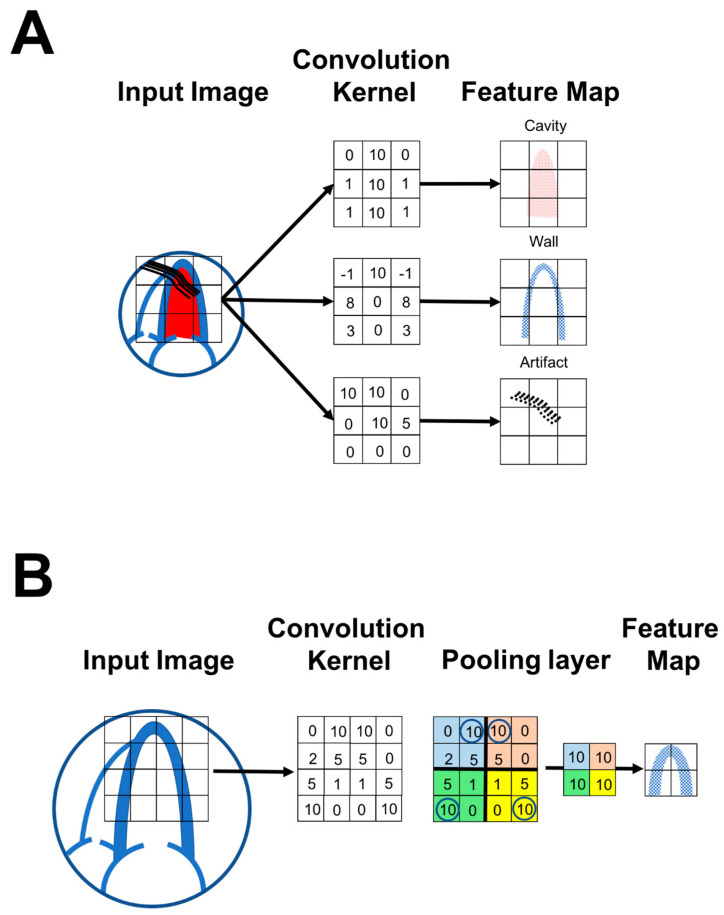
Convolution and pooling. (**A**) Convolution can separate intertwined information within the echocardiographic images by producing matrix kernels with specific features within the image. With repetition and model learning, the model can differentiate and separate several features within the image. For example, the kernels can distinguish left ventricular wall, cavity, and artifacts. (**B**) Pooling is a function that can be introduced in DL layers to reduce the processed data and prevent overfitting. Max pooling is a common type of pooling that reduces the convolution kernels to 2 × 2 matrices that contain the largest values in each part of the kernel. As a result, the output image is smaller and carries only the largest numbers from that specific piece within the image.

*Convolution:* convolution (i.e., building up complex features that can be derived via sliding across kernels—very good for edge detection) is one of the most important concepts in DL and a major differentiator from traditional ML algorithms. Simply, the concept of convolution deals with mixed information of different significance and meaning within the same dataset. For example, convolutional methods are commonly used to untwine different elements of an image for purposes of model learning. Convolution is used heavily in the fields of physics and engineering to simplify complex equations. In the field of imaging, convolution can be used to separate elements of the image or to identify distracting information in images. For example, in an echocardiographic image, convolution can identify and avoid artifacts during the training process of a DL model (Figure 4A). There is a multitude of complex mathematical methods available for convolution, and it remains unknown which interpretation of convolution fits best for DL. However, the cross-correlation interpretation method is considered currently the most useful method. The simple matrix is a digital representation of the pattern for detection of a specific feature. The output of the simple matrix is the altered image, which is often called a feature map. There will be one feature map for each element in the image. This process can be done by patching the image and panning this patch throughout the image until further processing is not possible. 

*Pooling:* pooling is a DL function that is interpolated between convolution layers with the purpose of reducing the amount of data being processed, preventing overfitting, and focusing on desired variables. There are several types of pooling; however, the most commonly used one is called ‘max pooling’. For example, if a 4 × 4 kernel matrix was derived from a source image, pooling would simply divide the 4 × 4 matrix into four 2 × 2 matrices and then take the largest number in each of these 2 × 2 matrices to produce a final 2 × 2 matrix with the largest numbers only. As the result, the output image would be smaller and would carry only the largest numbers from that specific piece within the image (Figure 4B). 

### 2.3. Examples of DL Models and Their Usability for Echocardiography 

In echocardiography, examples of applications of MLPs include presence and severity of diastolic dysfunction (present or absent, and grade I, II, and III), estimation of left ventricular ejection fraction (preserved or reduced), and wall motion score index. More importantly, an MLP can be used as a unit for building more sophisticated DL algorithms. 

Another popular DL algorithm is an autoencoder (AE). An AE is an unsupervised neural network that can reduce data dimensions by removing the noise in the data. The structure of the AE is based on MLP design (input, output, and hidden layers that function in a feedforward fashion); however, the AE generates an output that is as close as possible to its original input in an unsupervised manner. As such, the AE is suitable for feature learning, dimension reduction, and outlier detection. In echocardiography, an AE can be used for identification of LV end-systolic/diastolic frames in a moving echocardiographic video. It can also be used to identify myocardial speckle patterns. An AE can be also used with CNN and RNN (described below) in a hybrid model [35]. In one study, an AE was used for left ventricular segmentation, identification of end-systolic and end-diastolic volumes, and ejection fraction calculation based on 3D echocardiographic images [36]. 

**Figure 5:**
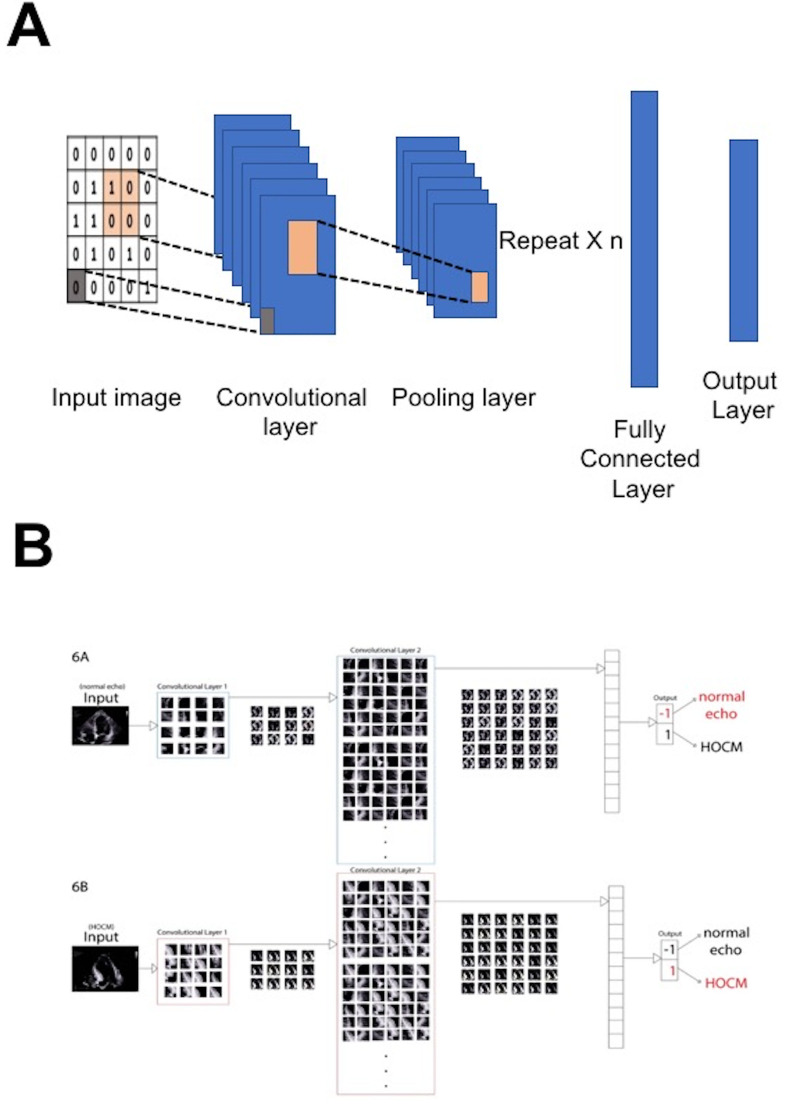
Convolutional neural networks (CNNs). (**A**) CNN algorithms are a feed forward neural network architecture, which is considered a significantly enhanced extension of an MLP, accomplished by inserting convolution layers. (**B**) An example of the application of a deep neural network in echocardiography, highlighting image processing to identify hypertrophic cardiomyopathy (HOCM) and differentiate it from normal.

The most popular DL algorithm in clinical research is the convolutional neural network (CNN, Figure 5), which is also an MLP design; however, the CNN represents an enhanced extension of an MLP achieved by inserting convolution layers at the level of the hidden layers. Such a design makes a CNN suitable for recognition of spatial data within the image [37,38]. A CNN can use spatial and anatomical echocardiographic data inputs to differentiate normal from abnormal patterns found in myocardial conditions that can be visually perceived similar, such as pathological and physiological LV hypertrophy. A pre-trained model is a model trained from one dataset followed by use of the parameters from this model to train another model on a different dataset. The pre-trained model can be applied for differentiation of other conditions (such as hypertrophic cardiomyopathy, infiltrative cardiomyopathy, or hypertensive heart disease) without the need to build a new model from scratch [39]. This method is an example of “transfer learning” [40]. However, transfer learning ultimately requires some degree of retraining or fine-tuning. Hybrid models composed of CNN architectures combined with other DL algorithms may be needed to capture spatial and acoustic information effectively within echocardiographic images, and, more importantly, to model temporal dynamics [41]. Recent studies used a modified CNN model to detect of wall motion abnormalities in both 2D and 3D images [42,43].

Madani et al. [44] utilized CNN architecture to classify 15 echocardiographic views, and found that DL can recognize these views with high accuracy (91.7%) compared with board-certified echocardiographers (70.2–84.0% accuracy). Zhang et al. [45] utilized CNN architecture to diagnose several cardiac conditions (i.e., hypertrophic cardiomyopathy, cardiac amyloidosis, and pulmonary arterial hypertension) using different echocardiographic views, and reported high c-statistics (0.85–0.93). Gao et al. [46] used CNN architecture for video classification of echocardiographic images that yielded classification accuracy up to 92.1%. Despite the impressive results, there are issues due to data and population differences. Strain analysis in echocardiography can be challenging [16]. Wang et al. used DL to perform strain analysis and found no significant difference relative to the traditional method in GLS measurement, but, even if a good performance was reached, the approach presented some limitations (e.g., most supervised methods heavily relied on large-scale synthetic datasets) [15]. Instead, Grenne et al. tried to overcome such limitations using custom-built DL-based ANNs specifically trained for motion estimation as an alternative to traditional speckle-tracking-based measures of strain. They found that, without any operator input, AI could perform motion estimation and measure GLS [14].

Recurrent neural networks (RNNs) represent another example of DL algorithms that have been used for sequence classification and video classification [47,48,49]. RNNs are unique compared with previously described algorithms since they have recurrent memory loops that can continuously process time series data, which allows sequencing inputs and events, while time series data may be difficult to process by MLP, AE, and CNN. This makes an RNN a suitable algorithm for analyzing temporal events within the echocardiographic images. In one study, a DL framework of RNNs was used for automatic characterization of cardiac cycle phases in echocardiographic images [50]. In another study, Abdi et al. [51] proposed a DL framework using RNNs to estimate the quality of echo cine videos from five different views and to provide feedback to the user in real time, with an average accuracy of 85%. The quality of the cine loop in the study was estimated from echo videos without pre-labeling [51]. Pandey et al. used a DeepNN classifier to assess diastolic dysfunction in patients with HFpEF who had elevated left ventricular filling pressures; however, even if good performance was reached, the authors did not perform external validation [52]. Instead, Tromp et al. tried to overcome such limitations using DL to assess diastolic function parameters. Most importantly, they performed external validation from different countries and healthcare systems, suggestive of generalizability [11].

Neural networks still need validation cohorts to calibrate the results, and clinical trials are needed before implementing in routine clinical practice. A generative adversarial network (GAN) is a particularly interesting application used for what is known as generative modeling. Generative modeling involves using a model to generate new examples from an existing distribution of samples. To achieve that, the model is trained using two neural network models, namely the “generator” and the “discriminator”. The generative network model learns to generate new plausible samples while the discriminative network model learns to differentiate generated examples from real examples. Both models continuously compete against each other in a process where the generator model seeks ‘to fool’ the discriminator. GANs have been used to generate photographs of non-existing human faces, to predict face aging, and to predict incidents and actions in videos. In echocardiography, GANs can help mitigate important problems of ultrasound images such as ultrasound dropouts and low-quality images. GANs can be also used for better and more realistic visualization in 3D echocardiography.

## 3. Supervised, Unsupervised, and Reinforced Deep Learning as Echocardiographic Solutions

Both DL and ML involve extraction of complex patterns within large datasets in supervised or unsupervised fashions [1]. In supervised learning, algorithms learn directly from large quantities of pre-labeled examples, i.e., the values of the output variable are known [8]. In the field of echocardiography, supervised algorithms have been used in 2D echocardiography to classify patterns of LV hypertrophy (physiological versus pathological hypertrophy) [44,53] and to identify constrictive pericarditis vs. restrictive cardiomyopathy [54]. Supervised algorithms have also been used for development of automatic systems for echocardiographic view classification [55,56,57], pediatric echocardiography classification [58], wall motion analysis [59], mitral valve leaflet segmentation [60], valvular heart disease classification [61], and ventricular function assessment [62]. 

**Figure 6:**
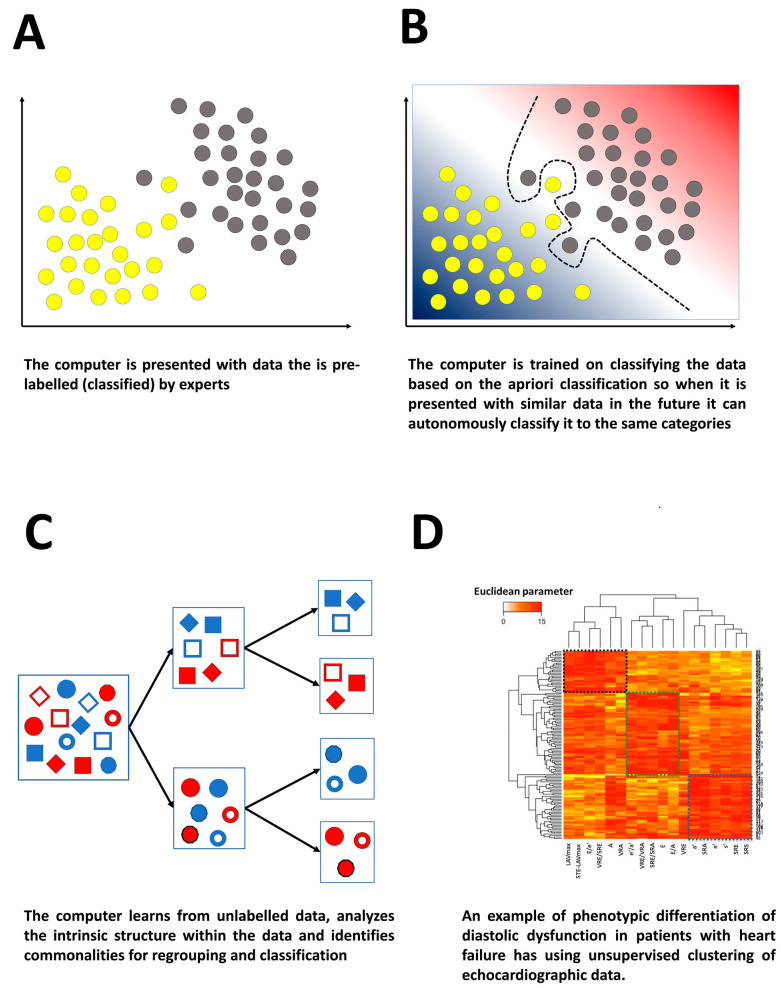
Supervised and unsupervised machine learning. (**A**,**B**) Supervised learning: (**A**) the computer is presented with pre-labeled data, and (**B**) the machine uses the a priori classification to separate the data so that they can be applied to unseen data without human interaction. (**C**,**D**) Unsupervised learning: (**C**) the computer is presented with unlabeled data and analyses the intrinsic structure and finds patterns used for re-grouping and reclassification, and (**D**) example of unsupervised clustering of patients using several conventional and deformational variables yielding three different clusters with distinct diastolic and LV functional properties.

Unsupervised learning, on the other hand, derives patterns from unlabeled data, i.e., the values of the output variable are not known. A common example of unsupervised learning is cluster analysis, where a dataset, without a priori knowledge of its true labels, is partitioned into clusters of ‘similar’ objects. Cluster analysis in medicine is a promising tool for mapping disease phenotypes (phenomapping) [63,64]. Unsupervised algorithms have also been introduced in echocardiographic research for discovering new disease subclasses [65]. Cluster analysis, or phenomapping, has been used to identify new groupings in several conditions such as coronary artery disease [65], left ventricular hypertrophy [66], acute heart failure [67], diabetes treatment [68], HfpEF [69], obesity [70], hypertension [71], and obstructive sleep apnea [72]. Recently, we have used cluster analyses for the diagnosis and characterization of subclasses of diastolic dysfunction from conventional and deformational echocardiographic variables (Figure 6) [73].

Reinforcement learning is another concept that can also be used in AI. In human psychology, learning by reinforcement focuses on promoting specific behaviors using reward (positive reinforcement) and punishment (negative reinforcement). In reinforced learning, software programs can act in a pre-specified environment to identify an appropriate behavior using “reward criteria” to influence the outcome of DL or ML models [27,74]. Based on decisions, these algorithms are penalized or rewarded, and by doing so they can maximize the accuracy of a model using trial and error. As such, reinforcement learning algorithms perform progressively better with training in ambiguous, real-life environments when choosing from an arbitrary number of possible actions, making them potentially fit for several clinical problems, including complex clinical imaging data. However, to date, reinforcement learning algorithms have not had much success in echocardiographic research.

## 4. Computer Vision and Video Classification

Computer vision operationalizes machines to recognize and analyze still images and videos. Recent advances in DL and computational capabilities have improved software abilities in video classification problems [75].

Video is an interesting classification problem because it includes both spatial (each frame holds important information) and temporal (the context of a frame relative to the frames before it in time) features. There are two main research areas on the comprehension of videos: video classification and video captioning. Video classification focuses on automatically labeling videos based on a collection of frames [76]. Basically, the algorithms dissect and classify video contents frame by frame as images and connect them together [77]. Video captioning generates short descriptions for videos and captures dynamic information such as human actions and car trajectories [78]. Unlike image classification, video classification has sequential frame input. The basic elements of echocardiographic data are similar to any ordinary video, making the application of video classification and captioning possible. In echocardiographic language, video classification and captioning are responsible for identification and labeling of different structures in motion (with time as a parameter) and their outlines within the image in different frames (e.g., the left ventricle and the left atrium, endocardial borders, etc.), as well as capturing different geometrical and deformational properties of these dynamic structures (e.g., the LV in relation to other structures as it contracts and relaxes). 

Echocardiographic videos pose a simpler learning problem relative to many other video classification tasks because all structures are the same within subsequent frames. To simplify this further, one can imagine a video that captures moving people on the street. With changing frames, the existing people continue to change and the positional relationship of each person to others is also continuously changing. If the recording camera position is also dynamic, the structures around the moving people, like buildings, street signs, traffic lights, etc., also change. Such layered and complex changes within the video makes predictions of subsequent events extremely difficult. In an echocardiographic video, however, the same structures continue to exist with fixed anatomical relationships and have repeated dynamic movements throughout fixed recorded frames in fixed time frames, and, as such, are predictable throughout the recorded video.

## 5. The Promised Future of the Echocardiographic Laboratory Is (Somewhat) Already Here

**Figure 7:**
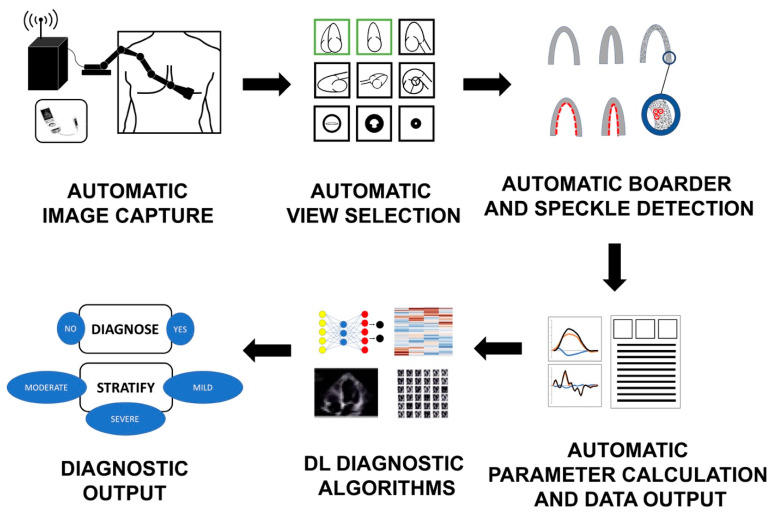
Ability of machine learning and deep learning to preform ordinary tasks of the echocardiography laboratory. Deep learning algorithms promise a great relief in preforming everyday ordinary tasks in an echocardiography laboratory, from image acquisition to diagnostic output. First, DL algorithms can be used to assist the use of handheld machines as well as the upcoming robotic ultrasound arms for both automatic and remote image capture. DL algorithms can assist novice learners in identifying whether the captured image is sufficient both in its quality and anatomic position. Second, DL algorithms can help in automatic identification of appropriate views and frames needed for calculation of specific measures such as the ejection fraction. Once these views are identified, the computer can automatically perform tasks such as endocardial border detection and speckle tracking for the production of parameters of volume and ejection fraction, and myocardial mechanics such as strain and strain rate. Hypothetically, DL algorithms can also be applied to obtain and calculate Doppler-derived parameters. After all parameters are obtained, other machine learning algorithms can be used to generate visual outputs such as curves, bull’s eyes, and measurement reports. At this stage, AI can help standardize subjective tasks such as wall motion analysis. The magnitude of the data output can be next used to generate other supervised and unsupervised AI algorithms suitable for diagnosis and classification, such as cluster analysis and neural networks. At this stage, AI algorithms promise exploration of new, previously unknown, disease subclasses.

Hypothetically, DL algorithms that can replace almost all ordinary tasks preformed in an echocardiographic laboratory already exist (Figure 7). The frontier has already been pushed for echocardiographic view recognition and echocardiographic variable quantification. For example, a DL-based algorithm that provides fully automated clip selection and calculation of the LV ejection fraction has been developed and validated. Recent studies have even tested the use of DL for complete automated interpretation of echocardiographic images [79,79,80]. Such a big step paves the way for other algorithms in equally needed, yet more debatable and variable areas of, echocardiographic interpretation that have long been dependent on subjective methods such as assessment of regional myocardial function and calculation of the wall motion score index at rest and at peak exercise. Automatic speckle tracking algorithms can also help to calculate differential strain measures, which is a task requiring high levels of human training and expertise both in acquisition and interpretation. 

DL can also serve as a diagnostic tool to differentiate physiological from pathological patterns, aid in deferential diagnosis, and help separate the “look-alike” diseases, and to classify, grade, and stratify disease processes. Examples include the appreciation of the presence and severity of diastolic dysfunction (present or absent, and grade I, II, and III); grading of the left ventricular ejection fraction (preserved, mid-range, or reduced); differentiation between constrictive versus restrictive pathology patterns; pathological and physiological LV hypertrophy diagnosis; assessment of hypertrophic cardiomyopathy, infiltrative cardiomyopathy, or hypertensive heart disease; and the diagnosis and assessment of severity of similar forms of vascular diseases.

Importantly, DL can serve as an add-on diagnostic tool in handheld and point-of-care ultrasound examinations and for medical robotic arms aimed at automated and remote acquisition and interpretation of echocardiographic images. DL can also be used as a powerful teaching tool aiding novice echocardiographers and imaging fellows, helping to mitigate learning curves and standardize the teaching process. 

Moreover, while most of the previous application examples are based on the current understanding and knowledge of cardiovascular diseases, perhaps the most important future promise of ML and DL is their ability to detect hidden patterns within the data and images that are not yet known (e.g., DL analysis for retinal images). Such discoveries will operationalize the field of phenomapping and discovery of new disease subclasses. This advancement is especially important in cardiovascular diseases that carry a great deal of complexity in both understanding their pathophysiological attributes as well as their therapeutic options. One clear example of this need is heart failure with preserved ejection fraction.

## 6. A “No-Contact” Echocardiographic Laboratory Model in the Next Emerging Pandemics

The technological revolution in medicine is probably more needed now than ever for future outbreaks. The cardiac patient of the future, wired with a network of biosensors, wearable monitors, and implantable miniature devices, investigated with robotic imaging arms and analyzed by computers, will be extremely different from our current patients. The tremendous amount of personalized data and clinical responses to daily stimuli will be processed using personalized software built by DL and beyond to direct the patient or even act independently towards the next appropriate action. 

As such, one can now envision the future of the echocardiography laboratory in a no-contact environment from both a patient side and the healthcare provider side. It is not hard any more to imagine patients walking in the echocardiographic laboratory where they are scanned by a device for personalized information and data, collected by all the wired devices, followed by complete no-contact echocardiographic procedures done autonomously by AI algorithms with minimal human interaction (Figure 7). First, AI algorithms are used to assist a robotic ultrasound arm for both automatic and remote image capture. Second, AI algorithms automatically identify the appropriate views and frames needed for calculation of specific measures. Once these views are identified, the computer automatically performs tasks such as endocardial border and wall speckle tracking to produce parameters of volume, ejection fraction, and myocardial mechanics such as strain and strain rate. After all parameters are obtained, ML algorithms are used to generate visual outputs such as curves and bull’s eyes, as well as measurement reports. The magnitude of generated data can be used in validated supervised and unsupervised AI algorithms for personalized diagnosis and classification of new disease subclasses. The final outcome is production of a definite personalized answer for a disease presence or absence, and its specific severity, and the suggestion of specific personalized therapeutic options. Importantly, this no-contact model would be applied in other areas of cardiac inpatient and outpatient services.

## 7. Current Challenge and Future Directions 

The implementation of robust DL in echo software could potentially augment clinical-decision making using a reliable automated assessment of video clips, static images, Doppler recordings, and speckle-tracking-derived information [27,41,81]. Although DL holds great promise in automating medical diagnosis, several challenges do exist and must be addressed and resolved before DL-based diagnostic algorithms can be applied in clinical practice. First, the currently available DL techniques remain poorly explored in echocardiography. More importantly, while many proposed architectures of DL exist for the use with public databases, not many of these are suitable for echocardiography. CNN, RNN, GRU, or LSTM (RNN variants) are commonly used but their practicality depends on optimization, activation functions, and features in the architectures. More studies are needed to test the feasibility and accuracy of the commonly used DL algorithms in echocardiography and to identify the algorithms more fit to be used with echocardiographic data. It is important to highlight that efforts to leverage DL algorithms fit for the field of echocardiography may not be exclusively the job of data scientists and programmers. There have been several efforts to introduce ML and DL platforms that are non-coder friendly and that can be immediately used without high-level training or detailed understanding of data science. Although clinicians are allowed to experiment with such platforms to uncover clinically relevant concepts, they should realize the complexities of applications of these pre-fetched platforms and work closely with data engineers to find specific solutions.

Second, DL requires massive amounts of pre-labeled data for training computers in the quest to achieve human-level classification performance. This is a clear distinction in performance between DL and traditional ML techniques as both differ as the scale of data increases: when using small datasets, DL algorithms tend to perform poorly compared with traditional ML algorithms. Although this leaves the field open for traditional ML techniques in echocardiography research, it is important to note that the type and amount of data suitable for training DL algorithms exist but frequently face the obstacle of healthcare privacy laws and medical data regulations, making medical data less available compared with other fields of computer science. The development of a homogenous nationwide echo database using standardized measures (e.g., the same vendors, protocol, and enhancer agents) and calibrated algorithms can be useful in that regard. In addition, a nationwide echo database in collaboration with echo vendors/software companies could promote research for algorithms suitable for heterogeneous echocardiographic databases and could potentially address these challenges, facilitating the application of DL in clinical decision-making.

Third, another set of challenges include the ability to process such massive amounts of data resulting from the variability of vendors, operators, software versions, and acquisition techniques, which can confound image processing. There is a need for high computational powers (e.g., quantum computation) to classify image details and moving images [82,83].

Fourth, technical aspects such as multiple variable optimization [84], artifact problems [85,86], poor acoustic window [45], focal feature localization evaluation methodology [87], or multiple focal feature detection [88] can be challenging in image recognition. There are proposed methodologies to address these problems (e.g., attention mechanisms) [89,90]. Moreover, further improvements in image segmentation algorithms are needed [91,92,93,94]. 

Fifth, lack of standardized approaches to DL challenge routine implementation of DL techniques. Examples include the choice of the learning rates [95] or differences in the results between max pooling and average pooling [96]. Along the same lines, the implementation of DL in echocardiography requires the identification of a universal clear stepwise workflow, from acquiring the image towards achieving a diagnostic or predictive output. 

Sixth, although DL as an application of AI that can be of most value in classification problems and pattern recognition, cognition problems are not exclusively classification problems. For example, DL or any other AI application has limited abstract reasoning. Connecting the dots in a picture drawn by a computer, and understanding and reasoning around the findings from a computer algorithm remain largely a human task. In a clinical universe, specifically in an echocardiographic world, and particularly in the next emerging pandemics, this may translate into less hand work, such as “image acquisition, parameter measurement, output exporting, etc.”; less human error and variability associated with these tasks; more patient and physician comfort; “less travel time, and fewer busy clinics, hospital schedules, and no-contact exams”; faster learning curves of novice cardiologists through dedicated DL educational algorithms; and more effective use of the human intellect to understand disease processes through observations and hypothesis generation. Finally, there is a significant potential bias in DL in healthcare. Thus, we need more representation of people of all backgrounds in clinical practice to train on such data.

## 8. Conclusions

In the era of advance computational power, utilization of big data analytics and DL in echocardiographic research promises reduction in cost, cognitive errors, and the intra- and inter-observer variability. Most importantly, the application of these techniques is of maximum importance in a projected “no-contact” medical service in future infectious outbreaks. However, several challenges still exist in both the clinical arena and the computer science field for the application of computer vision and DL in echocardiography. Overall, three key components are required to implement DL in echocardiographic imaging successfully: (1) improved architecture design for algorithms to be more compatible with echocardiographic data, (2) increased computational powers (e.g., quantum computation) to shorten the analytical process and improve predictive ability, and (3) generation of large amounts of echo data from individuals of all backgrounds with the ability to homogenize the data and alleviate variability.

## Figures and Tables

**Table 1:** Comparison between machine learning and deep learning.

	Machine Learning	Deep Learning
Description	Automated algorithms that progressively learn from data feed to make decisions and build predictive models ML can undertake tasks such as classification but it may be better in the context of a clinical review to avoid any statement that might be misinterpreted as implying that it can make decisions that relate to management	Interpretation of data relationships and features using multilayered data processing of neural network systems inspired from the human brain
Amount of data needed	A few thousand	A few million
Need for intervention by analyst	Need to examine variables within the data	Not needed as algorithms are self-directed towards relevant
Overfitting	Less likely with suitable amount of data (usually small amount of data)	More likely given the rarity of big data composed of millions of points
Outputs	Numerical (score, class)	Numerical (score, class) or non-numerical (various forms including elements, free text, sound, etc.)

**Table 2:** Basic deep learning algorithms, their optimal tasks, and their application in echocardiography.

Algorithm	Description	Optimal Task(s)	Examples in Echo
MLP	- Supervised classifications - Unit for other algorithms - Input and output vectors are not the same	Binary Classification	Assessing the presence of diastolic dysfunction
AE	- Unsupervised classifications - Feed forward only (no memory loops) - Input and output vectors are the same	Feature learning	- LV segmentation - LV end-systolic/diastolic volumes and EF - myocardial speckle patterns
CNN	- MLPs with convolutional layers - Feed forward only (no memory loops) - Most commonly used in clinical research - Transfer learning: output of one model can be applied to similar conditions	Spatial detail data recognition	Differentiation between normal and abnormal myocardial patterns (e.g., pathological and physiological hypertrophy)
RNN	- MLPs with memory loops - Memory loops (can loop data forward and backward compared with forward-only CNN)	Sequence classification	Automatic characterization of cardiac cycle phases in echocardiographic images
Hybrid models	Combinations of different DL algorithms used to refine results compared with single algorithms		Assessment of chamber size, wall thickness, regional LV function, and RV systolic pressure

## Data Availability

Not applicable.
